# Mumps Complications and Effects of Mumps Vaccination, England and Wales, 2002–2006

**DOI:** 10.3201/eid1704.101461

**Published:** 2011-04

**Authors:** Chee-Fu Yung, Nick Andrews, Antoaneta Bukasa, Kevin E. Brown, Mary Ramsay

**Affiliations:** Authors affiliation: Health Protection Agency Centre for Infections, London, UK

**Keywords:** Mumps, vaccination, hospital, orchitis, complication, viruses, England, Wales, CME, research

## Abstract

We analyzed data from hospital admissions and enhanced mumps surveillance to assess mumps complications during the largest mumps outbreak in England and Wales, 2004–2005, and their association with mumps vaccination. When compared with nonoutbreak periods, the outbreak was associated with a clear increase in hospitalized patients with orchitis, meningitis, and pancreatitis. Routine mumps surveillance and hospital data showed that 6.1% of estimated mumps patients were hospitalized, 4.4% had orchitis, 0.35% meningitis, and 0.33% pancreatitis. Enhanced surveillance data showed 2.9% of mumps patients were hospitalized, 6.1% had orchitis, 0.3% had meningitis, and 0.25% had pancreatitis. Risk was reduced for hospitalization (odds ratio [OR] 0.54, 95% confidence interval [CI] 0.43–0.68), mumps orchitis (OR 0.72, 95% CI 0.56–0.93) and mumps meningitis (OR 0.28, 95% CI 0.14–0.56) when patient had received 1 dose of measles, mumps, and rubella vaccine. The protective effect of vaccination on disease severity is critical in assessing the total effects of current and future mumps control strategies.

Mumps is an acute viral infection that is asymptomatic in ≈30% of children (*1*). Symptoms and signs include fever, headache, and swelling of the parotid glands, which may be unilateral or bilateral. Complications of mumps include orchitis, aseptic meningitis, oophoritis, pancreatitis, and encephalitis (*2**–**4*). Long-term complications include unilateral sensorineural deafness in children (*5*). To date, reported data on mumps complications are based on studies conducted mainly during the prevaccine era. Mumps vaccination was introduced into the UK immunization program as a single-dose mumps, measles, and rubella vaccine (MMR) for children 12 to 15 months of age in October 1988. The first vaccines contained the Urabe strain but this was changed to the Jeryl-Lynn strain in 1992 because of an unacceptable risk for aseptic meningitis (*1*). In 1996, to provide additional protection against all 3 infections, a second dose was added to the schedule. In the first decade after the MMR was introduced, rates of reported and confirmed mumps virus infection fell to extremely low levels in the United Kingdom. For persons born in the first 10 years of the program (1988–1998), vaccination coverage reached >90% for the first dose and ≈75% for the second dose of MMR by 5 years of age (*6*). Vaccine effectiveness in the UK has been estimated to be 87.8% for 1 dose and 94.6% for 2 doses of vaccine (*7*).

Since 1998, however, several mumps outbreaks have occurred in adolescents and young adults; these culminated in a national epidemic, mainly affecting university students, in 2004 and 2005. Clinical notifications of mumps increased from 4,203 in 2003 to 16,436 in 2004. The attack rate by birth rate was highest in those born between 1983 and 1986, with a rate of infection ranging from 140 to 170 per 100,000 population (*8*). Persons in this cohort were not offered routine childhood MMR and avoided mumps exposure because of high coverage in younger children. The rate of infection in persons born after 1988, and eligible to receive MMR, was substantially lower, and only 2.4% occurred in age groups eligible for 2 doses of MMR (*8*). Recent mumps surveillance data in England and Wales are showing an increase in the proportion of mumps cases in cohorts who should have received the 2-dose MMR (*9*). Two-dose MMR coverage in these cohorts has been estimated as ≈75% (*10*). Resurgences of mumps in vaccinated populations (including those who received 2-dose MMR) have been described in educational settings in other countries (*11**–**15*). Declining protection over time, and possible antigenic differences between the vaccine and outbreak strains, have been suggested as contributory factors (*7**,**16**,**17*). In the absence of natural boosting, therefore, future mumps epidemics may be unavoidable in vaccinated populations living in crowded, semiclosed settings such as colleges (*18*).

Because mumps is more severe in adults, increasing numbers of mumps cases in young adults in the postvaccine era could be expected to lead to a high rate of complications. A better understanding of mumps complication in vaccinated persons will therefore be essential in developing appropriate strategies to control mumps. We investigated hospitalizations associated with the mumps epidemic in England and Wales in 2004–2005 and used enhanced surveillance to compare the rate of complications among patients with confirmed mumps cases by age and vaccination status.

## Methods

We analyzed hospital episode, enhanced surveillance data, and clinical and laboratory surveillance data on mumps cases with onset or admission from April 1, 2002, through March 31, 2006, covering the period of the mumps outbreak in 2004–2005. When no onset date was available, the date of the sample or report was used.

### Hospital Episode Statistics

Hospital episode statistics (HES) capture all admissions to National Health Service (NHS) hospitals in England and Wales. The diagnoses recorded at the time of discharge are coded by using the International Classification of Diseases, 10th edition (ICD-10), and entered in any of 13 fields. A minimum dataset was extracted for all admissions with any of the following codes: B26 (mumps), N45 (orchitis and epididymitis), A87 (viral meningitis), N70 (oophoritis), and K85 (acute pancreatitis) (*19*). The anonymized HES identification field, generated from the NHS number, local patient identifier, postcode, sex, and date of birth, was used to link episodes from the same person admitted over the period (*20*).

### Enhanced Surveillance

In England and Wales, clinicians who diagnose mumps are required by statute to notify the proper officer for the local authority, usually a consultant in health protection. Since 1995, all notified cases of mumps have been monitored by offering oral fluid testing for immunoglobulin (Ig) M at the Centre for Infections, Health Protection Agency. A high proportion of cases are tested (50%–80%), and thus cases confirmed by testing for IgM in oral fluid provide data on the incidence of mumps (*1*). Vaccination history is requested on the sample-testing form for the oral fluid sample.

All patients with confirmed cases were then followed up by sending an enhanced surveillance form to the general practitioner (directly or through the local health protection unit) requesting further information. Information on complications, whether the mumps case-patient was hospitalized, and the receipt of MMR (or other mumps virus–containing vaccines) was confirmed. Those with no record of vaccination shown on the sample request form and in the general practitioner records (as noted on the returned enhanced surveillance forms) were classified as unvaccinated. Complications were recorded in free text, which was searched and recoded specifically for any mention of orchitis, meningitis, pancreatitis, and oophoritis.

### Estimating Total Mumps Cases from Laboratory-confirmed Mumps

Because of the high proportion of patients with notified cases that are tested by oral fluid, results for laboratory-confirmed mumps are thought to provide fairly complete estimates of clinically diagnosed mumps incidence. In 2005, however, during the peak of the mumps outbreak, mumps oral fluid testing was temporarily suspended in those born from 1981 through 1986. Therefore, to provide a better estimate of true incidence of clinically diagnosed mumps in 2005, we extracted the number of patients with clinically notified cases of mumps born during 1981–1986 from notifications of infectious diseases. In view of the high positive predictive value of clinical diagnosis in this age group and period, the number of clinically diagnosed patients with notified cases was then used as the total estimated denominator instead of laboratory-confirmed mumps cases.

### Statistical Analysis

Logistic regression analysis was used to assess the relation between hospitalization, mumps complications, and vaccination status in the enhanced surveillance data. The model was adjusted for age and sex, except in the model with mumps orchitis in which only male patients were included, and adjustment was made only for age. The age variable was included as a continuous variable by using polynomials up to the fifth degree to allow for nonlinearity of age. This option is an alternative to using a large number of age categories (which would give similar results). Odds ratios (ORs) with 95% confidence intervals (CIs) were determined; p<0.05 was considered significant. All statistical analysis was performed in Stata version 11 (StataCorp, College Station, TX, USA).

## Results

The total estimated number of mumps case-patients in England and Wales from April 1, 2002, through March 31, 2006, obtained by combining data from laboratory diagnosis and notifications (for those born between 1981 and 1986 in 2005 only), was 43,344 (23,246 male). A total of 2,647 mumps case-patients were hospitalized from April 2002 through March 2006 (Table 1). Hospitalized mumps case-patients, including those with a code for orchitis (996 patients), meningitis (154 patients), pancreatitis (146 patients), or none of these complications (1,418 patients) showed a clear increase during the outbreak (Figure). No hospitalized mumps case-patients also had been given a code for oophoritis. Most of these mumps complications were attributable to the mumps outbreak because episodes with these codes were negligible before the start of the outbreak period. Of mumps complications in hospitalized patients, most (81% of those with orchitis, 76% with meningitis, and 78% with pancreatitis) arose in those born from 1980 to 1989 (Table 1).

**Table 1:** Estimated proportion of mumps patients hospitalized and those given a code for complications by birth cohort, England and Wales, April 1, 2002–March 31, 2006*

Birth cohort	No. hospitalizations/total infections (%)	No. hospitalization complications/total infections (%)			
Orchitis*	Meningitis	Pancreatitis	None
Pre-1980	451/4,455 (10.1)	158/2,376 (6.7)	26/4,455 (0.6)	21/4,455 (0.5)	260/4,455 (5.8)
1980–1989	1,782/35,152 (5.1)	811/18,814 (4.3)	118/35,152 (0.3)	114/35,152 (0.3)	791/35,152 (2.3)
1990–2006	414/3,737 (11.1)	27/1,496 (1.8)	10/3,737 (0.3)	11/3,737 (0.3)	367/3,737 (9.8)
Overall	2,647/43,344 (6.1)	996/22,686 (4.4)	154/43,344 (0.4)	146/43,344 (0.3)	1,418/43,344 (3.3)

*In male patients >12 years of age only.

**Figure:**
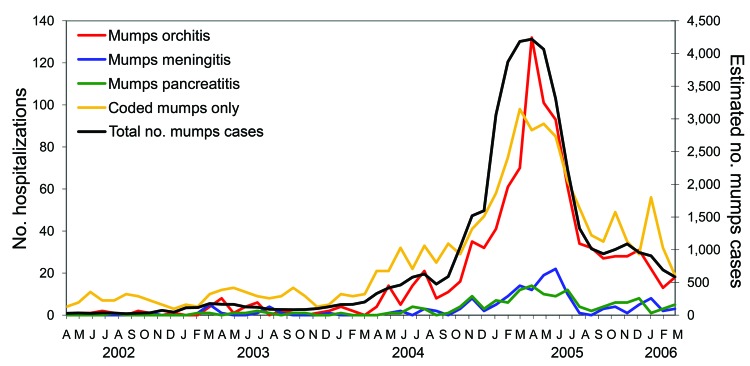
Total estimated number of cases of mumps and hospital episodes coded to mumps, England and Wales, April 1, 2002–March 31, 2006.

Therefore, the estimated rate of hospitalization was 6.1% (2,647/43,344) overall. The hospitalization rate was 4.4% (996/22,686) for mumps orchitis, 0.35% (154/43,344) for mumps meningitis and 0.33% (146/43,344) for mumps pancreatitis. When results are stratified by year of birth to those born before 1980, those born from 1980 through 1989, and those born from 1990 through 2006, the estimated complication rate for hospitalized mumps orchitis is lower in younger age groups than in older age groups (Table 1). Although rates of mumps meningitis and mumps pancreatitis in younger cohorts are lower, the pattern is less clear because of the smaller numbers involved. The rate of hospitalized mumps not coded with any of the main complications was lowest in the 1980–1989 cohort.

From April 2002 through March 2006, a total of 28,280 laboratory-confirmed mumps cases occurred. For 15,524 (55%) case-patients, the enhanced surveillance form was completed and returned. The response rate was higher in younger age cohorts. For those born pre-1980, the response rate was 52% (2,298/4,455), compared with 55% (10,865/19,763) for those born from 1980 through1989 and 68% (2,554/3,737) for those born from 1990 through 2006. For those born since 1990, the response rate was higher for confirmed case-patients listed as vaccinated on the sample request form (1765/2,189 [81%]) than for those with no vaccination details (789/1,548 [51%]).

Of 15,524 confirmed mumps case-patients whose enhanced surveillance form was returned, 7,226 (47%) had a documented history of vaccination (6,312 with 1 dose and 914 with 2 doses), and 8,298 (53.4%) were unvaccinated (many of whom were born before 1988 and therefore were not eligible for MMR). Hospitalization was noted for 452 (2.9%) mumps case-patients. The reported complication rate for mumps meningitis was 0.3% (53/15,524) and was 0.25% (38/15,524) for mumps pancreatitis. The most common complication was orchitis, reported for 6.1% (486/7,917) of male case-patients >12 years of age. The proportion of case-patients with each complication (excluding those with >1) that were hospitalized was lowest for mumps orchitis at 35.3% (166/470), followed by the proportion for mumps meningitis, 78.3% (36/46), and for mumps pancreatitis, 81.5% (22/27). The remaining 228 case-patients were hospitalized for varied reasons, ranging from airway concerns to anxious parents. The rate of hospitalization and rate of each main mumps complication were lower in those that were vaccinated than in the unvaccinated; rates were particularly low among those who had received 2 doses of vaccine (Table 2).

**Table 2:** Association between receipt of vaccination and mumps complications, adjusted for age and sex, England and Wales, April 1, 2002–March 31, 2006*

Complication	Vaccine dose	No. cases/total cases (%)	Unadjusted OR (95% CI)	Adjusted OR (95% CI)
Hospitalization	0	317/8,298 (3.8)	1	1
	1	122/6,312 (1.9)	0.50 (0.40–0.61)	0.54 (0.43–0.68)
	2	13/914 (1.4)	0.36 (0.21–0.64)	0.45 (0.25–0.80)
Orchitis†	0	356/4,574 (7.8)	1	1
	1	123/3,241 (3.8)	0.44 (0.36– 0.55)	0.72 (0.56–0.93)
	2	7/475 (1.5)	0.17 (0.08–0.37)	0.64 (0.28–1.44)
Meningitis	0	42/8,298 (0.5)	1	1
	1	10/6,312 (0.2)	0.31 (0.16–0.62)	0.28 (0.14–0.56)
	2	1/914 (0.1)	0.22 (0.03–1.57)	0.17 (0.02–1.26)
Pancreatitis	0	26/8,298 (0.3)	1	1
	1	12/6,312 (0.2)	0.61 (0.31–1.20)	0.95 (0.41–2.19)
	2	0/914	0 (0–1.34)‡	–

*OR, odds ratio; CI, confidence interval; –, not estimable. †Adjusted for age only. ‡Exact CI.

The ORs of reported hospitalization, orchitis, and meningitis were significantly lower in the vaccinated (1- or 2-dose MMR) than in the unvaccinated patients (Table 2). The polynomials for the age variable in the final logistic regression model for hospitalization, orchitis, meningitis. and pancreatitis are second, fourth, first, and second, respectively. Adjusting for age and sex had very little effect on the protective effect of vaccination in reducing the risk for hospitalization. The OR of having mumps meningitis was also found to be higher in male patients at 1.93 (95% CI 1.07–3.48) after vaccination status and age were controlled for.

## Discussion

The mumps outbreak in England and Wales led to a clear increase in hospitalizations caused by mumps complications, which mirrored the outbreak curve. From April 1, 2002, through March 31, 2006, the estimated hospitalization rate from HES data was 6.1% overall. A much lower rate of hospitalization (2.9%) was derived from the enhanced surveillance forms. In contrast, the rate of mumps orchitis estimated from HES data was lower than that found by enhanced surveillance. This may be explained by the fact that most mumps orchitis cases were managed in primary care. Most reported case-patients with mumps meningitis and pancreatitis were admitted to the hospital, but the estimated rate of these complications was low (<0.5%) by using either method.

On the basis of the rate of hospital episodes and data from enhanced surveillance, the complication rates observed here are low in comparison to results of studies from the prevaccine era. Previously published complication rates for mumps suggest that orchitis is the most common complication in 15%–30% of adult men with mumps (*21**–**24*). Mumps meningitis has been reported in 1%–10%, mumps pancreatitis in 4%, and mumps oophoritis in 5% of persons with mumps (*3**,**25**,**26*). The much lower rates observed in our study likely reflect the fact that the denominator is derived from population-based surveillance which aims to capture all cases of diagnosed mumps. Because the United Kingdom provides free universal access to primary care, we were able to ascertain milder cases that may not have been included in studies that use secondary care data or in studies conducted in other countries.

The estimated complication rates were lower in younger persons, particularly in the cohorts eligible for mumps vaccination. The outbreak in England and Wales during 2004–2005 affected mainly those born from 1980 through 1989 (*1**,**8*). Only those born in the second half of the 1980s could have been offered MMR; either routinely in the second year of life (those born from 1987 onwards) or as a catch-up at school entry for those who had not received measles vaccine. Those born after 1989 were eligible for routine MMR at 13 months and for a second dose of MMR at school age when it was introduced in 1996. The lower estimated hospitalization rate for mumps orchititis in younger cohorts could be attributed to less severe disease in younger persons or to the effect of mumps vaccination. The latter explanation was supported by the finding that a history of mumps vaccination was also associated with a lower risk for mumps hospitalization, mumps orchitis, and mumps meningitis in the enhanced surveillance data, even after age and sex were controlled for. Our analysis suggests that the adjusted odds of being hospitalized with mumps are reduced ≈50% in those with a history of at least 1 mumps vaccination. We observed an even lower rate of hospitalization in those who had received 2 doses than in those who had received 1 dose of vaccine, although this difference was not significant. Male patients had a higher risk for mumps meningitis, even after vaccination status was adjusted for. Results of vaccine effectiveness studies and the long-term persistence of mumps antibody have not shown differences on the basis of sex (*7**,**12**,**26*). However, mumps meningitis has been shown to affect male patients more often than female patients (*25*).

Most published complication rates derive from the prevaccine era; however, almost half of the case-patients included in our enhanced surveillance had been vaccinated. Our findings are more consistent with those of other studies in the MMR era in which rates of orchitis in postpubertal male patients were 10%–12%, and the rate of mumps meningitis was 0.9% (*11**,**12*). To our knowledge, information on the association between mumps vaccination and mumps complications is limited. A study of outbreaks of mumps in US colleges in 2006 showed no significant association between vaccination status and complications in a highly vaccinated population (*11*). The larger sample size in our analysis allowed us to detect differences in complication rates by vaccination status, which may be undetectable in smaller studies or when the number of unvaccinated persons is low. A limitation of the enhanced surveillance database is the possible bias from nonresponses. The higher response rates in younger, vaccinated persons would be expected to improve ascertainment of complications in this cohort. However, we observed lower complication rates in the young and vaccinated, which suggests that our observations are not due to response bias.

We believe it is plausible that vaccination against mumps can lead to a shift toward milder forms of the disease in a similar way as has been observed with varicella vaccine (*27*). Natural mumps in unvaccinated persons is known to be manifested as a minimally symptomatic infection with viral shedding (*28*). Studies have also reported a high proportion of asymptomatic or minimally symptomatic infections among vaccinated persons; more than half of case-patients did not have classical parotitis (*12*). The possibility of reduced severity of infections in vaccinated case-patients is also supported by findings of a lower virus isolation rate and shorter duration of viral detection in studies that compare vaccinated to unvaccinated patients (*29**,**30*). The lower rates of complications in vaccinated teenagers and young adults are consistent with secondary vaccine failure, which suggests that the primed person is able to mount an immune response to prevent more serious complications. A large number of cases with secondary vaccine failure is also consistent with declining protection with time since vaccination (*7**,**31*).

By using HES data, however, we could have underestimated the rates of complications because a substantial number of hospitalizations were coded for mumps alone. The overall rate based on hospital episodes is probably a high estimate because the numerator derives from an exhaustive database, whereas the denominator was derived from number of confirmed cases, a category that is prone to some underreporting. To minimize this underreporting effect, we combined clinical notifications during a period of high positive predictive value with laboratory-confirmed mumps cases derived from population-based surveillance by using noninvasive oral fluid testing. The use of laboratory-confirmed mumps cases based on serologic testing alone in the denominator is likely to overrepresent hospitalized case-patients and therefore to overestimate complication rates. The rate from enhanced surveillance is more likely to be a true reflection of absolute rate because both numerator and denominator are derived from the same source. In addition, although both estimates are dependent on patients seeking care for the complication, clinical details in the enhanced surveillance were supplied directly from primary care physicians who had diagnosed mumps. Therefore, complications exhibited some time after infection were less likely to be attributed to mumps.

The effects of long-term complications, such as sensorineural deafness and the possible link between mumps orchitis and infertility, were not included in our analysis (*3**,**32**–**35*). With the current outbreaks in colleges as well as in other congregate settings, mumps orchitis in postpubertal young men may require further research. A concern exists that mumps epididymitis (which carries a risk for testicular damage with subsequent infertility) is easily misdiagnosed as orchitis (*36*). As reports of mumps outbreaks in highly immunized populations of older teenagers and young adults continue to occur, the long-term effects of mumps complications may be substantial. Our analysis, however, suggests that vaccination provides higher levels of protection against hospitalization and risk for orchitis and meningitis in those diagnosed with mumps. The effect of vaccination on mumps complications will therefore be increasingly critical in assessing the outcome of current and future mumps control strategies.
